# Troponin utilization in patients presenting with atrial fibrillation/flutter to the emergency department: retrospective chart review

**DOI:** 10.1186/1865-1380-4-25

**Published:** 2011-06-08

**Authors:** Nazanin Meshkat, Emily Austin, Rahim Moineddin, Hamidreza Hatamabadi, Behzad Hassani, Ali Abdalvand, Amanda Marcuzzi

**Affiliations:** 1 Department of Medicine, Division of Emergency Medicine, University of Toronto, Toronto, Canada; 2 Queen's University, Kingston, Canada; 3 Department of Family and Community Medicine, University of Toronto, Toronto, Canada; 4 Division of Emergency Medicine, Shahid Beheshti University, Tehran, Iran; 5 University of Toronto, Toronto, Canada; 6 Shahid Beheshti University, Tehran, Iran; 7 York Central Hospital, Toronto, Canada

## Abstract

**Background:**

There are few recommendations about the use of cardiac markers in the investigation and management of atrial fibrillation/flutter. Currently, it is unknown how many patients with atrial fibrillation/flutter undergo troponin testing, and how positive troponin results are managed in the emergency department. We sought to look at the emergency department troponin utilization patterns.

**Methods:**

We performed a retrospective chart review of patients with atrial fibrillation/flutter presenting to the emergency department at three centers. Outcome measures included the rates of troponins ordered by emergency doctors, number of positive troponins, and those with positive troponins treated as acute coronary syndrome (ACS) by consulting services.

**Results:**

Four hundred fifty-one charts were reviewed. A total of 388 (86%) of the patients had troponins ordered, 13.7% had positive results, and 4.9% were treated for ACS.

**Conclusions:**

Troponin tests are ordered in a high percentage of patients with atrial fibrillation/flutter presenting to emergency departments. Five percent of our total patient cohort was diagnosed as having acute coronary syndrome by consulting services.

## Introduction

The prevalence of atrial fibrillation is estimated at 0.1% among adults younger than 55 years, rising with age to 9% in those older than 80 [1,2], with clear implications in resource utilization by an aging population. It is estimated that the number of patients with atrial fibrillation will rise to about 5.6 million in 2050 from its current number of 2.3 million cases [3]. Atrial fibrillation is a resource-intensive arrhythmia as 70-80% of these patients are admitted during the course of their illness [4]. More importantly, a number of studies have shown that the hospitalization rates for atrial fibrillation have increased, up to 2-3 fold in the last 2 decades [5,6].

Though there are few recommendations about the use of cardiac markers in the investigation and management of atrial fibrillation [7,8], these tests are often ordered to diagnose myocardial infarction as the cause or consequence of the arrhythmia in patients with atrial fibrillation or flutter that present to emergency departments. Unnecessary use of cardiac markers may contribute to emergency department laboratory costs, delays in patient discharge if using serial tests, and unnecessary admission and cardiac investigations for positive troponins of unknown significance. Currently, it is unknown how many patients with atrial fibrillation/flutter undergo troponin testing and how positive troponin results are managed in the emergency department.

We sought to look at the rates of troponins ordered by emergency physicians, the rates of positive troponins, and how many of those with positive troponins were diagnosed as acute coronary syndrome by admitting services. To our knowledge, no studies have looked at the use of troponins in atrial fibrillation/flutter or the impact of positive troponins on the management of these patients.

## Methods

### Patient population

A retrospective chart review was conducted at three Canadian centers: two academic emergency departments (AED 1 and 2) and one community emergency department (CED). A list of consecutive patients with new onset, paroxysmal or permanent atrial fibrillation, or atrial flutter that presented to the emergency departments during a 12-month period between January and December 2008 were generated by the health records departments at each center. All patients with the primary and secondary diagnosis of atrial fibrillation and atrial flutter were included. Exclusion criteria were as follows: patients presenting for INR checks and those directly admitted to a consulting service.

Research ethics board approval was obtained at all three sites.

### Sample size

To aim for a 95% confidence interval with a width less than 10% in the estimation of the rates of troponin testing and those treated for acute coronary syndrome, we reviewed a total of 450 charts (150 charts at each site). The charts reviewed were selected by systematic sampling from the list generated by the health record departments (described above). Charts with missing data were deleted.

### Study protocol

Three blinded data abstractors (BH, EA, AM) were trained by the principal investigator to abstract data using a reference manual. Data were recorded in a formatted electronic database. A pilot chart review consisting of 15 charts per center was conducted prior to the study to streamline data extraction. The principal investigator performed periodic random checks, and meetings were scheduled to resolve any disputed data. The intra-rater reliability was determined using the Cohen's kappa rating, aiming for a minimum inter-rater reliability of 80%.

All three sites used Troponin I (cTnI) as their cardiac marker. Two of the sites used the Beckman Coulter TnI immunoassay, and one site used the Abbott Architect TnI immunoassay. The cutoff troponin levels used were the respective coefficient variations of 10% for the specific immunoassays.

### Outcome measures

We looked at the rate of troponin tests ordered by emergency doctors, those with positive troponins, and those diagnosed with acute coronary syndrome by the consulting services (defined as being given the diagnosis of acute coronary syndrome in the chart by the staff cardiologist or internist). Patients whose charts indicated positive troponins were reviewed to determine if alternate reasons for elevated troponin levels other than acute coronary syndrome had been documented. We also looked at discharge and referral patterns, emergency department (ED) visit length of stay, and total emergency department (ED) length of stay. ED visit length of stay was defined as the time the patient arrived in the ED until the time they were either discharged or referred by the emergency room physician. Total ED length of stay was defined as the time the patient arrived in the ED until the time they left the ED, either after discharge by the emergency physician or a consulting service, or after being admitted.

## Results

### Characteristics of study patients

Between January and December 2008 a total of 1,148 patients with atrial fibrillation/flutter were seen across the three sites. A total of 451 charts were retrospectively reviewed. Table 1 summarizes the patient characteristics.

**Table 1:** Study and patient population characteristics

	AED1	AED2	CED	Total
Total ED visits Jan 01 2008 - Dec 31 2008	75,500	57,300	63,000	195,800

Total visits with AF as 1° or 2° Dx	473	271	404	1,148

**Patient characteristics (%)**				

	*N *= 150	*N *= 151	*N *= 150	*N *= 451

**Age**				

< 50	29 (19.3)	22 (14.6)	18 (12.0)	69 (15.3)

51-60	27 (18)	30 (19.9)	16 (10.7)	73 (16.2)

61-74	27 (18.0)	39 (25.8)	45 (30.0)	111 (24.6)

≥ 75	67 (44.7)	60 (39.7)	71 (47.3)	198 (43.9)

**Gender**				

Male	89 (59.3)	98 (64.9)	72 (48.0)	259 (57.4)

Female	61 (40.7)	53 (35.1)	78 (52.0)	192 (42.6)

**Type of AF**				

New onset	51 (34.0)	51 (33.8)	36 (24.2)	138 (30.7)

Paroxysmal	92 (61.3)	93 (61.6)	94 (63.1)	279 (62.0)

Persistent	7 (4.7)	6 (4.0)	9 (6.0)	22 (4.9)

Unknown	0 (0)	1 (0.7)	10 (6.7)	11 (2.4)

**Duration of symptoms**				

< 48 h	91 (60.7)	93 (61.6)	100 (67.1)	284 (63.1)

48 h	56 (37.3)	51 (33.8)	33 (22.2)	140 (31.1)

Unknown	3 (2.0)	7 (4.6)	16 (10.7)	26 (5.8)

**Past medical history**				

History of CHF or EF < 35%	40 (26.7)	30 (19.9)	10 (6.7)	80 (17.9)

HTN	91 (60.7)	80 (53.0)	98 (65.8)	269 (59.8)

CAD	49 (32.7)	31 (20.5)	43 (28.9)	123 (27.3)

DM	20 (13.3)	19 (12.7)	30 (20.1)	69 (15.4)

Previous stroke/TIA	21 (14.0)	23 (15.2)	28 (18.8)	72 (16.0)

Prosthetic heart valve	11 (7.3)	7 (4.6)	5 (3.4)	23 (5.1)

CHADS2 Score*				

0	41 (23.7)	45 (29.8)	29 (19.3)	115 (25.5)

1	25 (16.7)	43 (28.5)	40 (26.7)	108 (23.9)

2	34 (22.7)	34 (22.5)	39 (26.0)	107 (23.7)

≥ 3	50 (33.3)	29 (19.2)	42 (28.0)	121 (26.8)

*1 point each for congestive heart failure, hypertension, age > 75 years, diabetes, and 2 points for transient ischemic attack or stroke

### Intra-rater reliability

The kappa statistics for troponin results, presentation of chest pain, altered mental status, heart rate, hypotension, and treatment for acute coronary syndrome by consulting services were 0.9, 1.0, 0.89, 0.89, 0.64 and 1.0, respectively.

### Pattern of troponin testing and results

A total of 388 (86%) of patients had at least one set of troponin ordered. In those, 13.7% had positive troponin results, and 4.9% were treated as ACS. Forty percent of the patients (184/451) were kept for two or more sets of troponins; of those, 26.6% (49/184) had positive troponins, and 9.7% (18/184) were treated for ACS by the consulting service (summarized in Table 2).

**Table 2:** Troponin testing and results

	Total
	*N *= 451

**At least one troponin ordered**	388 (86%)

Positive troponins	53 (13.7%)

	

Treated as ACS	19 (4.9%)

	

**≥ 2 Troponins ordered **	184 (40.8%)

Positive troponin	49 (26.6%)

Treated as ACS	18 (9.7%)

The median troponin value was 0.61 ug/L (0.77, 1.19, 0.58 ug/L, respectively). Of all patients with positive troponins, 76.7% had other *potential *diagnoses for a rise in troponin other than ACS (demand-related rise in troponin 35.7%, congestive heart failure 28.5%, renal failure 16.6%, sepsis14.3%, pneumonia 2.3%).

The percentage of patients with positive troponins who underwent in-hospital workup for acute coronary syndrome, such as stress testing and/or angiograms, was 28.3% (15/53), with positive results in 46.7% (7/15).

### Referral patterns and ED length of stay

A total of 55% of patients were referred to consulting services. Those with positive troponins were referred 98% of the time to consulting services. The median ED visit and ED total length of stay for two sets of troponins versus patients who were not kept for more than one set of troponins were 374 and 289.5 min, and 808 and 375.5 min, respectively.

## Conclusions

Our study identified that a high percentage of patients with atrial fibrillation/flutter is kept in the emergency department for troponin testing. Eighty-six percent of patients had at least one set of troponins ordered, and 40% were kept for two or more sets. Our result is similar to another study, where 42.7% of patients were kept for a "rule-out myocardial infarction" protocol consisting of three sets of CK-MB cardiac markers [9].

In a recent Canadian study, 16.7% (range 10-27%) of patients with recent onset atrial fibrillation were admitted from the emergency department [10]. Admission rates were higher (55%) in our patient population; however, our study included all patients with atrial fibrillation. In our study, 14.7% of all those with at least one set of troponins and 26.6% of those with two or more sets had positive troponins. Of importance, 98% of patients with positive troponins were referred on to consulting services; however, only a third were treated as ACS by consulting services (4.9% of the entire patient cohort). It is possible that patients with positive troponins who were *not *treated as ACS could have required admission for other reasons; however, our study did not account for that.

The findings of our study have important implications for clinical practice and resource utilization. Cardiac troponins, while relatively cardiac-specific, are not disease-specific and can be positive in the absence of infarction [11]. For example, in a study of 1,000 consecutive patients who presented to an urban emergency department with potential symptoms or signs of coronary ischemia, 45% of patients with significantly elevated troponin I levels had a final diagnosis other than ACS [12]. In our study, 79% of patients with a positive troponin had a *potential *alternative diagnosis for a rise in troponin other than ACS, corroborating findings from other studies [13]. Demand-related rise in troponin could account for 35.7% of cases, as has been seen in numerous other studies [14-17]. In a study of patients presenting with elevated TnI levels with subsequent normal coronary angiogram, tachyarrythmias were the cause of TnI release in 28% of cases [18]. Failure to consider these other potential causes of elevated troponin can lead to unnecessary and invasive cardiac investigations and resource utilization, which will become of more importance with the introduction of newer, more sensitive troponin assays [19]. While it was not within the scope of this study, our work raises interesting questions about the possibility of identifying patients with specific symptoms or characteristics who warrant further diagnostic workup versus patients who are safe to discharge home. These types of questions are best answered in a prospective study, for which our work sheds light on important characteristic in the study population.

Our study has a few limitations. There was no gold standard to establish the diagnosis of acute coronary syndrome, and since the diagnosis of acute coronary syndrome given by cardiologists (and internists) can be open to interpretation, we cannot say with certainty that those who were treated for ACS truly required the treatment. Further, due to the design of our study, we did not look at adverse events in patients who were and were not treated for ACS. There are also inherent limitations of a retrospective chart review, such as the lack of clinical homogeneity among the different sites, missing clinical data, and variability in data abstraction. Specifically, the intra-rater reliability was lower for hypotension compared to other variables, as reflected in the lower kappa statistic. This is likely related to the fact that while even the data abstraction protocols stated that the 'presenting' blood pressure be measured, we did not explicitly define that this blood pressure should be the triage blood pressure, and not any other record. This could have led to confusion regarding which blood pressure was actually recorded. Unfortunately, this was not picked up during our pilot study and random checks. Despite this, we feel our results are sufficiently robust given that a kappa of 0.64 still reflects "substantial agreement," and all other kappa values were high.

In summary, a high percentage of patients with atrial fibrillation/flutter are kept in Canadian emergency departments for troponin testing, and 4.9% of the total cohort was treated for acute coronary syndrome. Further prospective studies are required to study the clinical implications and prognosis of patients with positive troponins in patients with atrial fibrillation/flutter and to identify those patients who should be tested for troponin and be treated for acute coronary syndrome.

## Competing interests

The authors declare that they have no competing interests.

## Authors' contributions

NM conceived the study, designed the trial, obtained funding, supervised the conduct of the trial and data collection, and drafted the manuscript. EA was a data abstractor, helped with literature review, and edited the first draft of the manuscript. RM provided statistical advice and analyzed the data. HH helped with data abstraction. AA helped with data abstraction. BH and AM were data abstractors.
